# The G Protein-Coupled Bile Acid Receptor TGR5 (Gpbar1) Modulates Endothelin-1 Signaling in Liver

**DOI:** 10.3390/cells8111467

**Published:** 2019-11-19

**Authors:** Caroline Klindt, Maria Reich, Birte Hellwig, Jan Stindt, Jörg Rahnenführer, Jan G. Hengstler, Karl Köhrer, Kristina Schoonjans, Dieter Häussinger, Verena Keitel

**Affiliations:** 1 Department of Gastroenterology, Hepatology and Infectious Diseases, University Hospital, Medical Faculty of Heinrich Heine University Düsseldorf, 40225 Düsseldorf, Germany; caroline.klindt@med.uni-duesseldorf.de (C.K.); Maria.reich@uni-duesseldorf.de (M.R.); Jan.stindt@uni-duesseldorf.de (J.S.); haeussin@uni-duesseldorf.de (D.H.); 2 Department of Statistics, TU Dortmund University, 44221 Dortmund, Germany; hellwig@statistik.tu-dortmund.de (B.H.); rahnenfuehrer@statistik.tu-dortmund.de (J.R.); 3 Leibniz Research Centre for Working Environment and Human Factors, TU Dortmund, 44139 Dortmund, Germany; hengstler@ifado.de; 4 Genomics and Transcriptomics Laboratory, Biologisch-Medizinisches-Forschungszentrum (BMFZ), Heinrich Heine University Düsseldorf, 40225 Düsseldorf, Germany; Koehrer@uni-duesseldorf.de; 5 Laboratory of Metabolic Signaling, École Polytechnique Fédérale de Lausanne, CH-1015 Lausanne, Switzerland; kristina.schoonjans@epfl.ch

**Keywords:** bile acids, endothelin-1, sinusoidal endothelial cells, hepatic stellate cells, portal hypertension

## Abstract

TGR5 (Gpbar1) is a G protein-coupled receptor responsive to bile acids (BAs), which is expressed in different non-parenchymal cells of the liver, including biliary epithelial cells, liver-resident macrophages, sinusoidal endothelial cells (LSECs), and activated hepatic stellate cells (HSCs). Mice with targeted deletion of TGR5 are more susceptible towards cholestatic liver injury induced by cholic acid-feeding and bile duct ligation, resulting in a reduced proliferative response and increased liver injury. Conjugated lithocholic acid (LCA) represents the most potent TGR5 BA ligand and LCA-feeding has been used as a model to rapidly induce severe cholestatic liver injury in mice. Thus, TGR5 knockout (KO) mice and wildtype (WT) littermates were fed a diet supplemented with 1% LCA for 84 h. Liver injury and gene expression changes induced by the LCA diet revealed an enrichment of pathways associated with inflammation, proliferation, and matrix remodeling. Knockout of TGR5 in mice caused upregulation of endothelin-1 (ET-1) expression in the livers. Analysis of TGR5-dependent ET-1 signaling in isolated LSECs and HSCs demonstrated that TGR5 activation reduces ET-1 expression and secretion from LSECs and triggers internalization of the ET-1 receptor in HSCs, dampening ET-1 responsiveness. Thus, we identified two independent mechanisms by which TGR5 inhibits ET-1 signaling and modulates portal pressure.

## 1. Introduction

TGR5 (Gpbar1) is a G protein-coupled bile acid receptor expressed in various cell types, including macrophages, as well as non-parenchymal liver cells such as activated hepatic stellate cells (HSCs) and liver sinusoidal endothelial cells (LSECs) [1,2,3,4,5]. Activation of TGR5 occurs after binding of bile acids (BAs), leading to an intracellular increase of cyclic AMP (cAMP) as second messenger and to the activation of further downstream signaling [6,7,8]. TGR5 is known to play an important role in biliary epithelial cell function and has anti-inflammatory as well as cytoprotective properties [2,4,6,9,10,11,12,13,14]. TGR5 knockout mice (TGR5 KO) have a very mild phenotype without any signs of overt liver disease [15,16]. However, TGR5 KO are more susceptible towards cholestatic and inflammatory liver injury [17,18,19]. While TGR5 is responsive to all human primary and secondary BAs, irrespective of conjugation state, the secondary BA taurolithocholic acid (TLC) represents the most potent TGR5 BA agonist [6,20]. Interestingly, feeding wildtype (WT) mice a diet enriched in 1% (*w*/*w*) LCA triggers within a few days segmental bile duct obstruction by crystal precipitation leading to the development of focal areas of necrosis (bile infarcts) with subsequent recruitment and accumulation of neutrophils, as well as to the induction of periportal fibrosis [21,22]. Using mice deficient for either the intercellular adhesion molecule-1 (ICAM-1 KO mice) or the catalytic subunit of NADPH oxidase (gp91phox KO mice), it was demonstrated that the contribution of neutrophils to liver injury following LCA feeding is negligible. Thus, the hepatotoxicity stems from LCA and its metabolites directly, and this model can be used to rapidly induce severe cholestatic liver injury, which also shows characteristics of sclerosing cholangitis [21,22].

The burden of liver diseases continues to rise in European countries and accounts for approximately 150,000 deaths per year attributed to liver disease, of which about two-thirds of patients die before the age of 65 years [23]. The most important non-malignant complication of chronic liver disease in humans is the development of portal hypertension (PH). PH results from increased intrahepatic vascular resistance, which is due to structural changes within the sinusoids during fibrosis development, to dysfunction of HSCs and LSECs as well as to microvascular thrombosis and platelet dysfunction [24,25]. Nitric oxide (NO) is an essential regulator of portal pressure. However, NO release from LSECs is decreased in cirrhotic livers, contributing to LSEC dysfunction [24]. We have previously demonstrated that TGR5 is expressed in LSECs, where activation of the receptor induced expression and activation of endothelial NO synthase (eNOS) and subsequent generation of NO [1].

While TGR5 was not expressed in quiescent HSCs in vitro and in vivo, cultivation of isolated HSCs on plastic dishes, which triggers differentiation into a myofibroblast-like phenotype (activated HSCs), led to an upregulation of TGR5 mRNA and protein levels [4,26]. These findings suggest that BA signaling via TGR5 may play a role in the regulation of hepatic vascular tone and portal pressure under physiological but also disease conditions. Since LCA feeding not only resulted in severe cholestatic liver injury and recruitment of inflammatory cells, but also rapid development of periductal fibrosis [21], we studied the impact of short-term LCA diet supplementation in TGR5 WT and KO mice.

## 2. Materials and Methods

### 2.1. Materials

Dulbecco’s modified Eagle Medium (DMEM) was from Gibco (Thermo Fisher Scientific, Waltham, MA, USA). FCS was acquired from Perbio Science (Bonn, Germany). Pencillin, streptomycin, and amphotericin B were purchased from Gibco (Thermo Fisher). Taurolithocholic acid (TLC) and forskolin were purchased from Sigma-Aldrich (St. Louis, MO, USA). DMSO (Dimethyl sulfoxide) was acquired from Carl Roth (Karlsruhe, Germany). The TGR5 agonist RO5527239((R,E)-1-(4-(3-(hydroxyimino)-3-(2-methylpyridin-4-yl)-1-o-tolylpropyl)phenyl)piperi-dine-4-carboxylic acid) was kindly provided by F. Hoffmann-La Roche, (Basel, Switzerland) [27]. Pronase P and Dnase I for isolation of murine HSCs were also from F. Hoffmann-La Roche. Pronase E for isolation of HSCs was purchased from Sigma-Aldrich and collagenase type I from Worthington (Lakewood, WA, USA). Collagenase type I for isolation of LSECs was from Sigma-Aldrich.

### 2.2. Animal Experiments

TGR5 transgenic mice were a generous gift from K. Schoonjans and J. Auwerx and have been shown to overexpress TGR5 in different tissues, including liver [28]. TGR5 knockout (KO) mice were kept on a C57BL/6 background and were kindly provided by the Schering-Plough Research-Institute (Kenilworth, NJ, USA) [15,29]. Heterozygous animals were used for breeding to obtain littermate TGR5 knockout and wildtype animals. Mice were bred and kept in the central animal facility of Düsseldorf University (ZETT) and had access to water and food ad libitum. A 12 h light/dark cycle was maintained. TGR5 KO and WT male mice aged 8–12 weeks were fed a diet containing 1% (*w*/*w*) lithocholic acid (LCA, ssniff, Soest, Germany) for 84 h or standard chow diet ad libitum. After 84 h of feeding, mice were anesthetized by intraperitoneal injection of ketamine/sodium pentobarbital solution. Blood was collected and anesthetized animals were subjected to portal vein perfusion prior to collection of liver tissue. All animal experiments were approved by the local authorities (LANUV).

### 2.3. Serum Analysis

Serum samples of WT and TGR5 KO mice were taken at the end of the experiment. Serum was analyzed for AST and ALT using the Spotchem Analyzer (Axon Lab AG, Reichenbach, Germany).

### 2.4. Determination of Hepatic Hydroxyproline Content

Hydroxyproline content was determined from liver tissue (TGR5 KO and WT) according to I.S. Jamall [30].

### 2.5. Isolation of Hepatic Stellate Cells

HSCs were isolated from 4–6 month-old female WT C57BL/6 mice. Mice were kept under the same conditions as described above. Mice were anesthetized and placed under a heating lamp. After cannulation of the portal vein, the liver was perfused using HBSS buffer (37 °C). Following a perfusion with pronase E (0.2% in HBSS buffer) and collagenase type I (0.025% in HBSS buffer), that led to digestion of collagen and lysis of hepatocytes, the liver was removed and mechanically diced. The liver cell solution was filtered and resuspended in a pronase/Dnase solution (0.125%) for further digestion. After filtration through a 70 μm cell strainer, the solution was diluted in HBSS and centrifuged at 500× *g* for 7 min. The supernatant containing cell debris was removed. The cell pellet was resuspended in HBSS containing 0.25% BSA and then mixed with 28.7% Nycodenz gradient solution (Cosmo bio USA, Carlsbad, CA, USA) to obtain a final concentration of 18% Nycodenz (*w*/*v*). This preparation was covered with 8 mL 0.25% BSA/HBSS before centrifugation for 30 min at 1400× *g* without brake. After centrifugation, the layer between the two buffers was harvested and centrifuged at 450× *g* for 10 min. The supernatant was discarded and the pellet was resuspended in DMEM supplemented with 10% fetal calf serum (FCS) and 1% penicillin/streptomycin/amphotericin B. The medium was exchanged every other day. Cells were kept on cell culture plates covered with collagen type 1 in a density of 2.5–3 × 10^6^ cells/well in a 6-well plate. Cells were used for experiments on the next day or after seven days for experiments with activated HSCs (Supplemental Figure S1). During experiments, cells were kept in DMEM without FCS or antibiotics.

### 2.6. Isolation and Cultivation of LSECs

The protocol for magnetic-activated cell isolation of LSECs was adapted from [31]. After induction of anesthesia, a cannula was inserted into the portal vein for perfusion with HBSS containing collagenase type 1, leading to removal of blood and dissociation of liver cells from connective tissue. Afterwards, livers were removed, diced, and incubated in the collagenase solution for 20 min for further digestion. The cell suspension was filtered through 100 μm cell strainers and centrifuged several times for removal of collagen and cell debris. To clear the cell suspension of CD45-positive immune cells, the cell suspension was preincubated with anti-CD45 magnetic beads for 15 min at 4 °C and transferred onto LD columns in the QuadroMACS separator (Miltenyi Biotec GmbH, Bergisch Gladbach, Germany). The flow-through was centrifuged for 10 min at 500× *g* and 4 °C. The supernatant was discarded and the pellet was resuspended in ice-cold MACS-buffer, according to the manufacturer’s protocol. For positive selection of LSECs the cell suspension was incubated with anti-CD146 MicroBeads (Miltenyi Biotec). Then the cell suspension was put on cell columns in the OctoMACS separator (Miltenyi Biotec), according to protocol provided by the company. The flow-through was discarded and the columns were flushed with MACS-buffer containing 0.05% BSA after removal of columns from the magnetic field. The cell suspension was centrifuged and the pellet was resuspended in cell culture medium (DMEM, supplemented with 10% FCS and 1% antibiotics). Cells were cultured in cell culture dishes covered with collagen type I with the following densities: 24-well: 800,000–900,000 cells/well; and 6-well: 3–3.5 × 10^6^ cells/well. Culture medium was removed and cells were washed in PBS after 24 h in culture. Cells were treated with serum-free medium containing DMSO, 10 μM TGR5 agonist, 25 μM taurolithocholic acid, or 10 μM forskolin for 24 h.

### 2.7. Gene Expression Assay

RNA from liver samples of the above-mentioned mice or of LSECs was extracted using the Maxwell 16 LEV simply RNA Tissue Kit (Promega, Madison, WI, USA) and the Maxwell 16 Instrument (Promega, Madison, WI, USA), according to the manufacturer’s instructions.

A DNA microarray platform (Affymetrix) was used for global gene expression analysis.

Total RNA preparations were checked for RNA integrity by Agilent 2100 Bioanalyzer quality control. Mean RNA integrity number (RIN) was 7.3 ± 0.4 (range 6.5 to 8.1) for liver tissue from chow-fed animals and 7.8 ± 0.3 (range 6.5 to 8.8) for liver tissue from LCA-fed animals. RNA was further analyzed by photometric Nanodrop measurement and quantified by fluorometric Qubit RNA assays (Life Technologies).

Synthesis of biotin-labeled cDNA was performed according to the manufacturers’ protocol (WT Plus Reagent Kit; Affymetrix, Inc., Thermo Fisher Scientific, Waltham, MA, USA). Briefly, 100 ng of total RNA was converted to cDNA. After amplification by in vitro transcription and 2nd cycle synthesis, cDNA was fragmented and biotin labeled by terminal transferase. Finally, end-labeled cDNA was hybridized to Affymetrix Mouse Gene 2.0 ST Gene Expression Microarrays for 16 h at 45 °C, stained by strepatavidin/phycoerythrin conjugate and scanned as described in the manufacturers´ protocol. Data analyses on Affymetrix CEL files are described in Section 2.14—Statistical Analysis of Expression Data. The gene expression data can be downloaded from the NCBI GEO database (https://www.ncbi.nlm.nih.gov/geo/, accession number GSE139075).

For real-time PCR, 1 μg of this RNA was used to generate cDNA utilizing the QuantiTect Reverse Transcription Kit (Qiagen, Hilden, Germany). Gene expression was quantified using Taqman Gene Expression Assays (Thermo Fisher Scientific, Waltham, MA, USA, assay order information can be obtained upon request) and the Lightcycler 480 II (Roche Diagnostics, Rotkreuz, Switzerland). Data were produced in duplicates for each gene. Mean values of cycle numbers of the target gene were subtracted from the mean of cycle numbers of the house-keeping gene succinatdehydrogenase (SDHA) for the respective sample. These values taken to the power of 2 are the mRNA expression of the target genes in relation to SDHA expression. The number of independent experiments performed are given in the text/figure legends. At least three independent experiments were performed. In order to rule out a regulation of the chosen house-keeping gene SDHA in response to LCA feeding, we initially analyzed expression of selected genes in relation to SDHA, hypoxanthine phosphoriosyltransferase-1 (HPRT-1), and glyceraldehyde-3-phosphate dehydrogenase (GAPDH). Since no difference in regulation was observed between these house-keeping genes, we continued the experiments with SDHA, as shown in Table 1 and Figure 3.

### 2.8. ET-1 ELISA

For analysis of ET-1 secretion from LSECs, cells were kept in culture after isolation as described for 24 h. After washing of cells with PBS, they were treated with serum-free medium containing DMSO, 10 μM TGR5 agonist, 25 μM TLC, or 10 μM forskolin for 24 h. Then, the supernatant was collected and transferred onto amicon ultra-10 centrifugal filters (Merck Millipore, Burlington, NJ, USA) to concentrate the supernatant (1:10). The concentrated supernatant was analyzed for ET-1 levels using the Quantikine kit system for murine ET-1 (Research and Diagnostic Systems Inc, Minneapolis, MN, USA), according to the provided protocol. LSECs were washed and incubated with a fixed amount of 50 μL lysis buffer for protein isolation. Measurement of protein concentration in lysate was performed in the Multiskan Spectrum (Thermo Fisher) and ET-1 concentration in the supernatant was normalized relative to protein concentration.

### 2.9. HSC Contraction Assay

For quantification of activated HSC contraction, 6-well plates were covered with a thick layer of 1 mL/well rat tail collagen. Rat HSCs were isolated as described before [32]. Cells were put on 6-well plates at a density of 6 × 10^5^ cells/well. Cells were kept in DMEM supplemented with 10% FCS and 1% antibiotics for 7 days. Medium was exchanged every 48 h starting the day after isolation. At day 7 after isolation, cells were treated with serum-free medium containing DMSO, 10 μM TGR5 agonist, or 10 μM forskolin for 30 min. After detachment of collagen lattices from the 6-well surface, cells were treated with ET-1 (Sigma-Aldrich) at a concentration of 1nM to induce contraction for 2 h and 24 h. Photos of cell/collagen lattice areas were taken at the time of ET-1 addition and after 2 h and 24 h (Supplementary Figure S2). Surface area was measured using the ImageJ distribution Fiji V.2.0.0 [33,34]. The lattice area used as control treated with DMSO and vehicle for the respective time points was set to 1.0.

### 2.10. Measurement of Portal Pressure

TGR5 KO and WT mice on chow or LCA diet (1%, 84 h) were anesthetized and the portal vein was cannulated. Invasive blood pressure measurement was performed using the pressure transducer P75 and amplifier from Hugo Sachs Electronics (Harvard Apparatus GmbH, March, USA). Additionally, 6-week-old WT mice were anesthetized and cannulation of portal vein for blood pressure measurement was done. Mice livers were then perfused with either control HBSS solution or a solution containing 10 μM TGR5 agonist for 15 min. Subsequently, increasing concentrations of ET-1 (5, 10, and 15 nM) were added to the perfusion media for 15 min each. Perfusion pressure in the portal vein was measured constantly over the whole time course. Mean portal pressure was taken for each condition of each mouse. Portal pressure under control conditions (t = 0) of HBSS buffer perfusion supplemented with DMSO (=vehicle) were set to 1.0.

### 2.11. Hematoxylin Eosin- and Picro-Sirius Red Staining

Liver samples were fixed in 4% paraformaldehyde, embedded in paraffin, and sliced into sections of 5 μm. Following rehydration, liver slides were stained in hematoxylin (Hematoxylin Solution Gill No.3, Sigma Aldrich) for 1 min and in eosin for 3 min. Alternatively, slides were treated with the Picro-Sirius Red staining kit according to the manufacturer’s instructions (Polysciences, Warrington, FL, USA). After dehydration, slides were mounted with VectaMount (Vectorlabs, Burlingame, CA, USA). Images were acquired on a Zeiss AxioLab A1 Microscope (Carl Zeiss, Jena, Germany) with 63×, 40×, 20×, and 10× objectives.

### 2.12. Immunofluorescence of Cells in Culture

HSCs were isolated and kept in culture as described above. Seven days after isolation, cells were incubated with serum-free medium containing either DMSO, 10 μM TGR5 agonist, or 10 μM forskolin. After 24 h, cells were washed with PBS and incubated with 5 μg/mL wheat germ agglutinin (WGA) (Thermo Fisher) labeled with AlexaFluor-594 in PBS for 10 min at 37 °C to stain glycoproteins or glycolipids within the outer leaflet of the plasma membrane [35]. Cells were fixed with ice-cold methanol for 3 min after removal of the WGA-solution. After washing of cells, unspecific binding was blocked using a solution of 5% FCS in PBS for 30 min. Activated HSCs were treated with either primary antibodies for Endothelin-A receptor (Abcam, Cambridge, UK) (1:500) or just blocking solution as negative control for 1 h. A secondary antibody labeled with AlexaFluor-488 (Dianova, Hamburg, Germany) (1:100) was used for detection of the primary antibody and incubated for 1 h. LSECs were fixed with methanol and incubated with antibodies directed against vascular endothelial Cadherine (Santa Cruz Biotechnology, Dallas, USA) (1:100) and TGR5 (Gpbar1 8/50, Roche) (1:20). Secondary antibodies labeled with Cyanine-3 (Dianova) (1:500) or fluorescein (Dianova) (1:100) were used at dilutions of 1:500 and 1:100, respectively. Intranuclear DNA was labeled by Hoechst 34580 (Thermo Fisher) (1:20,000). Pictures of LSECs were taken using the confocal microscope LSM 510 (Carl Zeiss, Jena, Germany). HSCs were imaged using a LSM 810 confocal laser microscope (Carl Zeiss, Jena, Germany). Colocalization analysis of murine hepatic stellate cells was carried out using Pearson’s correlation coefficient calculated by the coloc2 plugin for ImageJ after selection of representative regions of interest ROIs in cell surface areas [33,34].

### 2.13. Statistical Evaluation

The data are presented as means and the standard error of the mean (SEM). Statistical significance was tested using the two-sided Student’s *t*-test or the Wilcoxon signed-ranked test as appropriate. A *p*-value < 0.05 was considered a significant result and a *p*-value < 0.01 was considered a highly significant result.

### 2.14. Statistical Analysis of Expression Data

Data preprocessing and all subsequent analyses were performed using the statistical programming language R, version 3.5.0 (R Development Core Team 2018). Normalization of the raw microarray data (CEL files) was done using RMA as implemented in the R package oligo. Normalization was performed on a set of, in total, 18 CEL files. To determine differentially expressed genes, the R package limma was used [36]. Adjustment for multiple testing was conducted with the method of Benjamini and Hochberg (FDR, false discovery rate) [37]. A gene was called differentially expressed if the adjusted *p*-value was <0.05 and log2 fold change was <−1.5 (downregulated) or >1.5 (upregulated). A volcano plot was generated that plots log2 fold change on the x-axis and statistical significance on the y-axis (-log10 of the FDR-adjusted *p*-value). Heatmaps were used to visualize z-scores (expression values standardized per gene to mean 0 and standard deviation 1), ordered according to average linkage hierarchical clustering of genes and experiments, respectively. Gene ontology enrichment analysis was performed based on probe set IDs with the topGO package [38], using Fisher’s exact test and the elim method. Only results from the biological process ontology were considered. The cutoff for the enrichment *p*-value was set to 0.05.

## 3. Results

### 3.1. LCA Feeding Results in a Significant Upregulation of Genes Associated with Cholestasis, Inflammation, and Extracellular Matrix Remodeling

**Figure 1:**
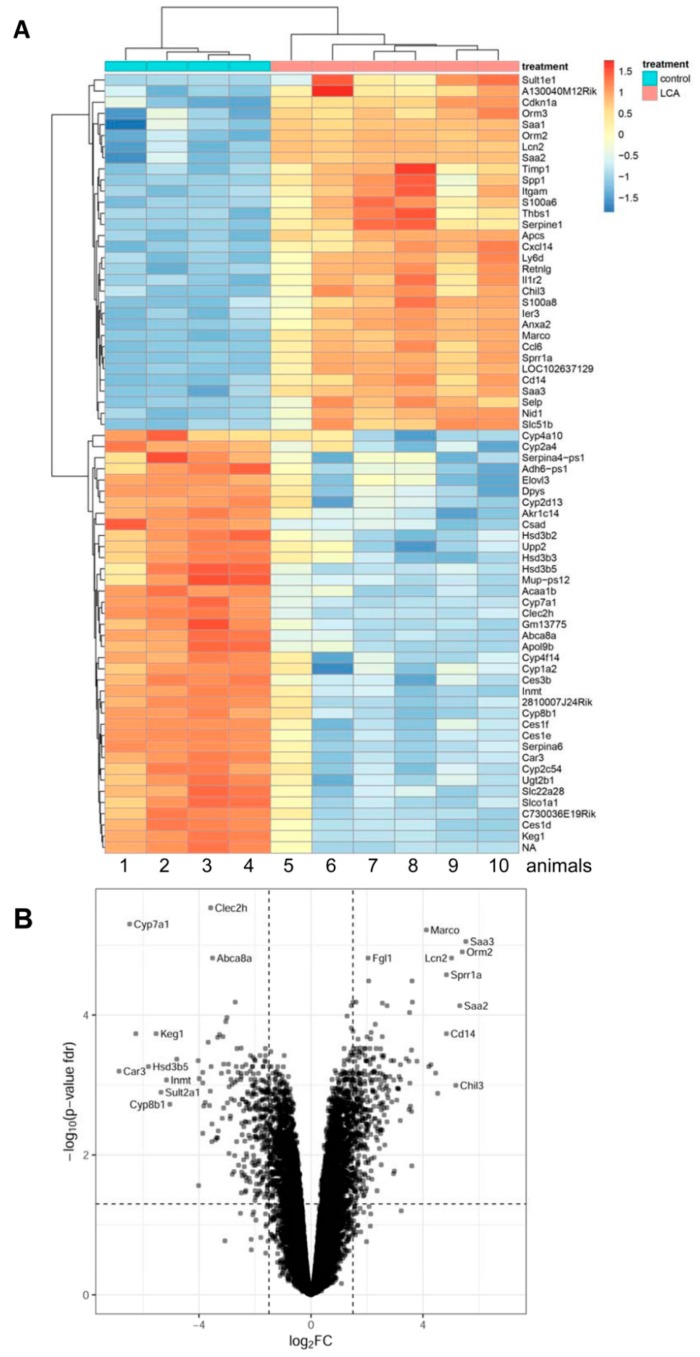
Influence of a chow diet supplemented with 1% lithocholic acid (LCA) over 3.5 days on gene expression in liver tissue of mice. (**A**) Heatmaps of z-scores (expression values standardized per gene to mean 0 and standard deviation 1) of the strongest differentially-regulated genes (*p* < 0.05 and log2 fold change >3 (upregulated) or <−3 (downregulated)). Each row corresponds to a gene, each column to a mouse. Rows and columns were ordered according to average linkage hierarchical clustering. (**B**) Volcano plot: On the x-axis, the log_2_ fold change is plotted, and on the y-axis, -log10 of the false discovery rate (FDR)-adjusted *p*-value is plotted.

Feeding of a chow diet supplemented with 1% LCA over 3.5 days (84 h) resulted in a severe cholestatic liver injury in wildtype (WT) mice, as described previously [21]. Liver tissue of LCA-fed WT mice was compared to chow-fed WT littermates by gene array analysis. In total, 332 and 263 genes were significantly up- or downregulated, respectively (*p* < 0.05; FDR-adjusted, and log2 fold change <−1.5 or >1.5; Supplementary Tables S1 and S2). Overrepresented Gene Ontology (GO) groups among the upregulated genes were associated with proliferation, inflammation, and extracellular matrix organization (Supplementary Tables S1 and S3, Figure 1). The four upregulated genes with the lowest *p*-values were the acute phase proteins serum amyloid A3 (Saa3) (46.2-fold up) and orosomucoid 2 (Orm2) (42.4-fold up), the bacterial siderophore binding protein lipocalin 2 (Lcn2) (32.4-fold up) that is expressed in neutrophils, and the pattern recognition receptor MARCO (macrophage receptor with collagenous structure, 17.4-fold up), which is found on macrophages and dendritic cells (Supplementary Table S1). The most significantly overrepresented GO groups among the downregulated genes were associated with oxidation-reduction processes, sodium-independent organic anion transport, and bile acid biosynthesis. The rate-limiting enzyme of bile acid synthesis, Cyp7a1 (89.0-fold down), was among the strongest downregulated genes (Supplementary Tables S2 and S4). Therefore, gene expression profiling of the livers of mice under LCA diet revealed a profile of an inflamed, cholestatic organ undergoing extracellular matrix reorganization [21,22].

We have previously reported that cholestatic liver injury in response to bile duct ligation is more severe in TGR5 knockout (KO) mice as compared to WT littermates [19]. Therefore, we analyzed liver damage following LCA feeding in TGR5 WT and TGR5 KO littermates.

### 3.2. Liver Damage in Response to LCA Feeding is Aggravated in TGR5 KO Mice and Results in Increased Portal Pressure

**Figure 2:**
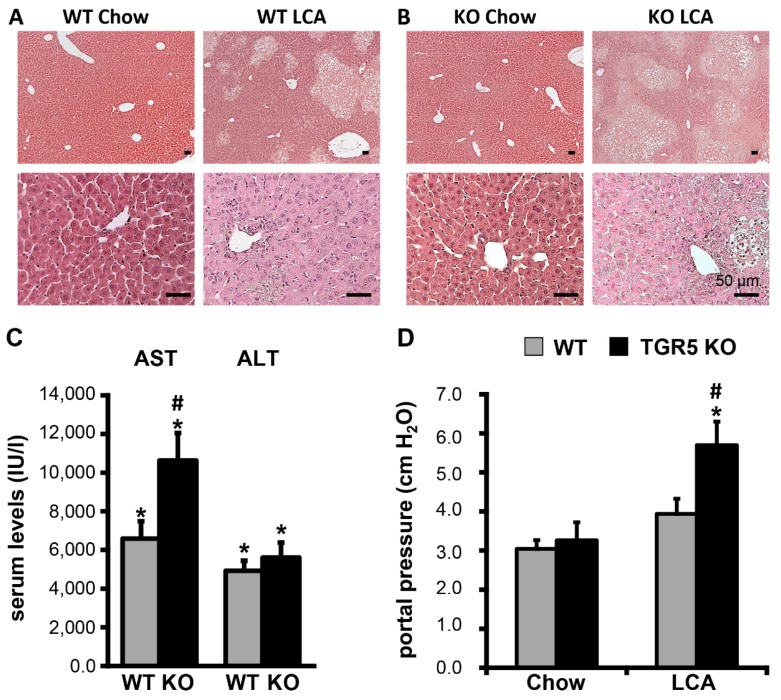
LCA feeding induces a more pronounced liver injury in TGR5 knockout mice as compared to littermate controls. (**A**) Hematoxylin and eosin staining of representative liver sections from wildtype mice fed with either a chow or LCA-enriched diet (1% *wt*/*wt*) for 84 h. (**B**) H&E staining of liver sections from TGR5 knockout (KO) mice. LCA feeding results in a more severe liver injury in TGR5 KO mice as compared to controls. Bars = 50 μm. (**C**) Serum AST and ALT-levels (in IU/l) increase significantly in mice of both genotypes following LCA feeding (*n* = 10–12 for LCA-fed and *n* = 4 for chow-fed mice of each genotype). (**D**) Measurement of portal vein perfusion pressure (in cm H_2_0) of wildtype (WT) and TGR5 KO mice fed with either chow or LCA diet (*n* = 5–7). All data are shown as mean ± standard error of the mean (SEM). * Statistically significant difference between the LCA- and chow-fed mice of the same genotype (*p* < 0.05); # statistically significant difference between WT and TGR5 KO mice fed the same diet (*p* < 0.05).

Liver damage in response to LCA feeding was more pronounced in TGR5 KO mice as compared to WT mice, as demonstrated by significantly higher serum levels of AST (WT LCA (*n* = 12) 6586 ± 898 IU/L vs. KO LCA (*n* = 10) 10,629 ± 1422 IU/L (1.6-fold increase), *p* < 0.05) and more extensive bile infarcts on liver histology (Figure 2A–C). Furthermore, a significant increase in portal venous pressure was observed in LCA-fed TGR5 KO mice as compared to LCA-fed WT or chow-fed TGR5 KO mice, respectively (WT LCA (*n* = 7) 3.9 ± 0.4 cmH_2_0 vs. KO LCA (*n* = 6) 5.7 ± 0.6 cmH_2_0 (1.4-fold increase), *p* < 0.05) (Figure 2D). There was no difference in portal pressure between chow-fed mice of both genotypes.

### 3.3. Genes Associated with HSC Activation are Significantly Upregulated in TGR5 KO Mice as Compared to WT Littermates

**Figure 3:**
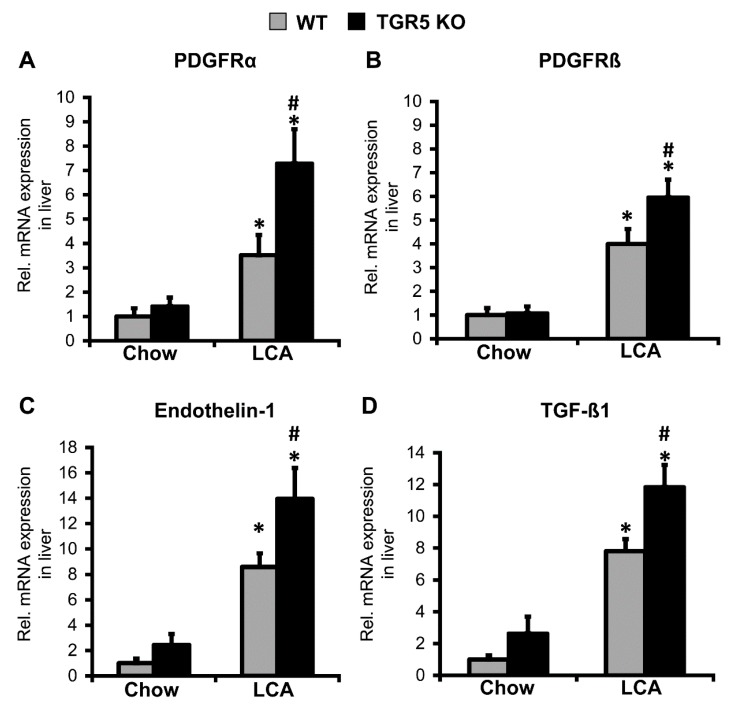
Expression of genes related to portal hypertension and fibrosis development are significantly higher in liver tissue of TGR5 KO mice as compared to WT littermates in response to LCA feeding. (**A**–**D**) Semi-quantitative real-time PCR analysis of liver tissue revealed an upregulation of mRNA expression PDGFRα, PDGRFβ, endothelin-1, and TGF-β1 following LCA feeding. The increase was more pronounced in mice deficient for TGR5. Data are presented as mean ± SEM (*n* = 4–12). * Statistically significant difference between the LCA- and chow-fed mice of the same genotype (*p* < 0.05); # statistically significant difference between WT and TGR5 KO mice fed the same diet (*p* < 0.05; using the two-sided Student’s *t*-test).

**Figure 4:**
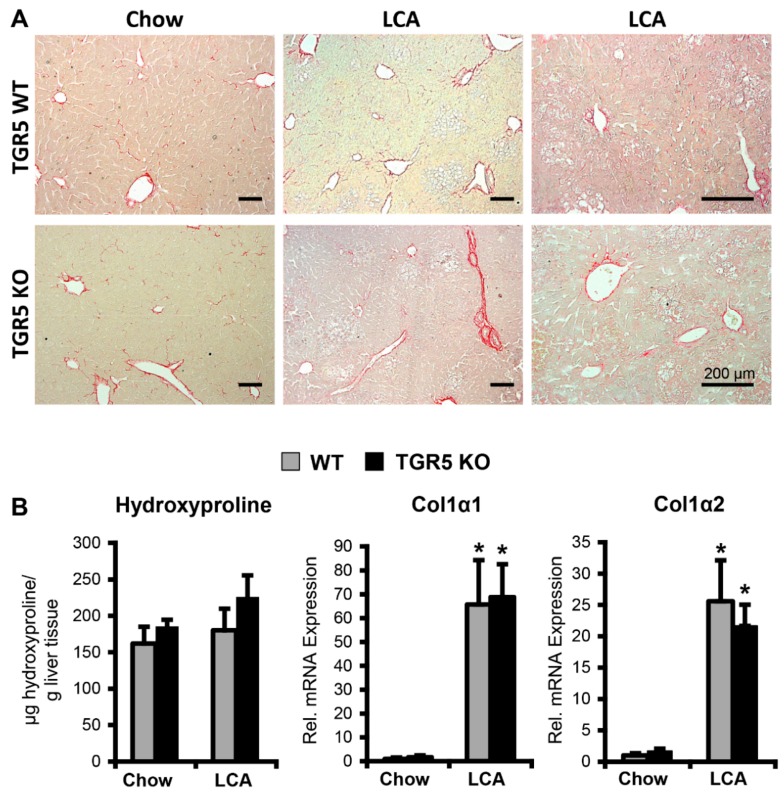
LCA-feeding triggers an increase in extracellular matrix production in both genotypes. (**A**) Representative Sirius red staining from one chow-fed and two LCA-fed animals of each genotype demonstrates a slight increase in fibrous tissue in both genotypes. Bars = 200 μm. (**B**) Measurement of hydroxyproline content in liver tissue showed a slight increase following LCA feeding, which did not reach significance (*n* = 8–10 animals per genotype LCA group, *n* = 3 animals per genotype for chow group). However, expression of collagen-1α1 and collagen-1α2 increased significantly after LCA feeding in both genotypes (qPCR*: n* = 12–14 LCA-fed animals per genotype, *n* = 4 chow-fed animals per genotype). Liver tissue from WT animals is shown in grey, while tissue from TGR5 KO mice is represented by black bars. All data are shown as mean ± SEM. * Statistically significant difference between the LCA- and chow-fed mice of the same genotype (*p* < 0.05) (*n* = 8–10).

Gene expression analysis by semi-quantitative real-time PCR revealed a similar pattern of gene regulation in both genotypes after LCA feeding. As expected, induction of cholestasis by LCA feeding resulted in a significant downregulation of Cyp7a1 and Cyp8b1, the rate limiting enzymes of bile acid synthesis. In line with the increase in liver damage, a significant upregulation of the genes encoding receptor-interacting serine/threonine-protein kinases 1 and 3 (RIPK1 and RIPK3), as well as caspase-3, was observed (Table 1). Furthermore, we found a significant induction of pro-inflammatory genes such as tumor necrosis factor-α (TNF-α), CCL2, interleukin-1β (IL-1β) and pro-fibrogenic chemokine receptors CCR1, CCR5 (Table 1) [21,22,39]. Interestingly, genes related to sinusoidal endothelial dysfunction, activation of HSCs, and fibrosis development, such as platelet-derived growth factor receptor-α (PDGFRα), PDGFRβ, transforming growth factor-β1 (TGF-β1), and endothelin-1 (ET-1), were upregulated in both WT and TGR5 KO mice following LCA feeding. However, the observed increase was significantly higher in TGR5 KO mice (PDGFRα: WT LCA (*n* = 7) 3.52 ± 0.8 vs. KO LCA (*n* = 10) 7.30 ± 1.4, 2.1-fold increase in TGR5 KO, *p* < 0.05; PDGFRβ: WT LCA (*n* = 12) 4.0 ± 0.6 vs. KO LCA (*n* = 12) 6.23 ± 0.75, 1.6-fold increase, *p* < 0.05; TGF-β1: WT LCA (*n* = 7) 7.81 ± 0.8 vs. KO LCA (*n* = 9) 11.8 ± 1.4, 1.5-fold increase, *p* < 0.05; ET-1: WT LCA (*n* = 14) 8.58 ± 1.1 vs. KO LCA (*n* = 11) 13.9 ± 2.4, 1.6-fold increase, *p* < 0.05) (Figure 3A–D). Sirius red staining of liver tissue after 3.5 days of LCA feeding revealed a slight increase in periportal collagen deposition, but no overt hepatic fibrosis in both genotypes, which was in line with measurements of hydroxyproline content in liver tissue (Figure 4A,B). Expression of genes encoding collagen-1α1, collagen-1α2 were upregulated in both genotypes following LCA feeding for 3.5 days, indicating initiation of extracellular matrix synthesis, as described earlier (Figure 4B) [21,22].

### 3.4. Activation of TGR5 in LSECs Lowers ET-1 Expression and Secretion

**Figure 5:**
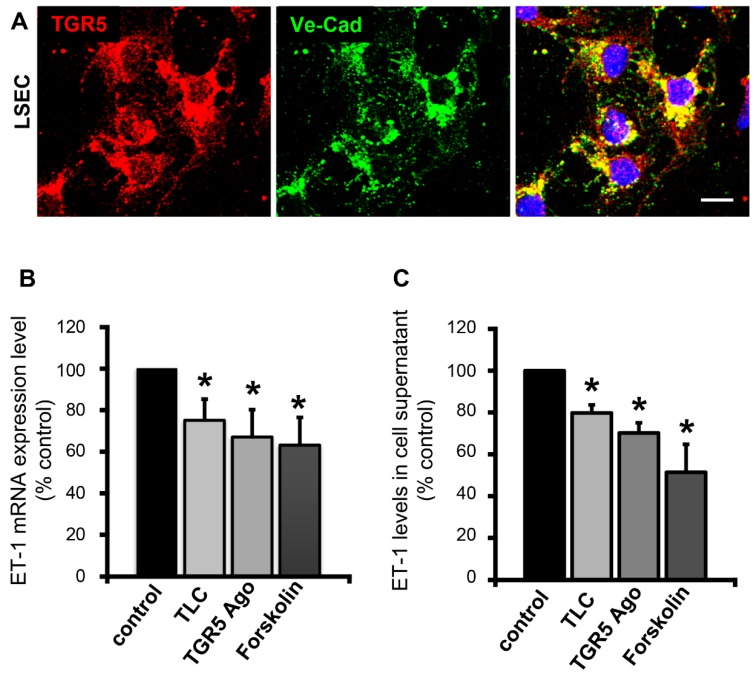
Stimulation of TGR5 in isolated liver sinusoidal endothelial cells (LSECs) reduces endothelin-1 (ET-1) mRNA expression and secretion. (**A**) Immunofluorescence staining of TGR5 in isolated LSECs (in red) demonstrates localization of the receptor within the plasma membrane, as well as in intracellular vesicular structures. An antibody against vascular endothelial cadherin (VE-cad, shown in green) was used to visualize cell junctions near the plasma membrane. Nuclei were stained with Hoechst (shown in blue). Bar = 10 μm. (**B**) ET-1 mRNA levels in isolated murine LSECs in response to a 24-h incubation with DMSO (=control), TLC (25 μM), a TGR5 agonist (10 μM), or forskolin (10 μM). The mRNA level after DMSO treatment was set to 100%. (**C**) Detection of ET-1 protein amounts by ELISA in the cell supernatant of LSECs incubated for 24 h with DMSO (=control), TLC (25 μM), a TGR5 agonist (10 μM), or forskolin (10 μM). ET-1 protein levels after stimulation with DMSO were set to 100%. Data are presented as mean ± SEM (n = 6–9). * Statistically significant difference as compared to DMSO-treated controls (*p* < 0.05).

ET-1 in liver is mainly secreted by LSECs under physiological conditions [24]. We have previously demonstrated that rat and human LSECs express TGR5 [1,4]. To determine whether secretion of ET-1 from LSECs was modulated by TGR5, LSECs were isolated from WT mice. Expression and localization of TGR5 in these primary cells was confirmed by immunofluorescence staining with an antibody against TGR5 and an antibody against vascular endothelial cadherin (Ve-Cad). TGR5 was localized in the plasma membrane, as well as in intracellular vesicular structures (Figure 5A). Treatment of primary murine LSECs with vehicle (DMSO), taurolithocholic acid (TLC, 25 µM), a non-bile acid TGR5 agonist (RO5527239, 10 µM) [27], and forskolin (10 µM) for 24 h resulted in a significant decrease in ET-1 mRNA expression (DMSO vs. TLC (*n* = 10) 1.0 vs. 0.76 ± 0.06 (1.3-fold reduction, *p* < 0.01); DMSO vs. TGR5 Ago (*n* = 7) 1.0 vs. 0.73 ± 0.08 (1.4-fold reduction, *p* < 0.05); DMSO vs. forskolin (*n* = 6) 1.0 vs. 0.58 ± 0.12 (1.7-fold reduction, *p* < 0.05)) (Figure 5B). We have previously demonstrated that stimulation of TGR5 in LSECs through coupling to a stimulatory G protein triggered an increase in intracellular cAMP [1]. Therefore, forskolin, a direct activator of adenylate cyclase, was used as TGR5-independent positive control [1]. Furthermore, detection of ET-1 in the cell culture supernatant of these cells demonstrated a reduction in ET-1 protein levels in response to TLC, the TGR5 Ago, and forskolin (Figure 5C) (DMSO vs. TLC (*n* = 10) 1.0 vs. 0.80 ± 0.05 (1.3-fold reduction, *p* < 0.01); DMSO vs. TGR5 Ago (*n* = 9) 1.0 vs. 0.67 ± 0.12 (1.5-fold reduction, *p* < 0.05); DMSO vs. forskolin (*n* = 5) 1.0 vs. 0.54 ± 0.11 (1.9-fold reduction, *p* < 0.05)). Thus, stimulation of TGR5 not only reduces ET-1 mRNA expression, but also ET-1 secretion from LSECs.

### 3.5. Activation of TGR5 in HSCs Attenuates ET-1-Mediated Contractility

**Figure 6:**
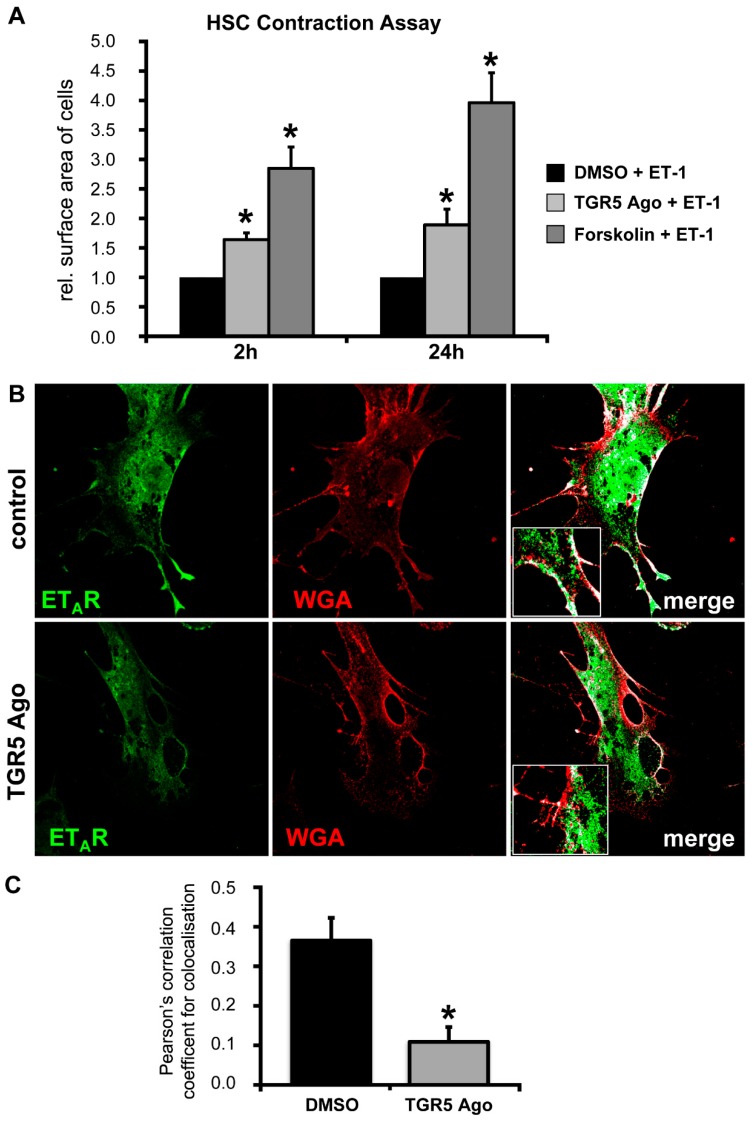
Stimulation of TGR5 in isolated HSCs triggers internalization of the endothelin A receptor (ET_A_R), thus reducing the contractile response towards ET-1. (**A**) Contraction of rat HSCs on collagen lattices was measured in response to ET-1 after pre-incubation with DMSO, a TGR5 agonist (10 μM), or forskolin (10 μM), which was used as TGR5-independent positive control. Surface area of collagen lattices served as indirect measure of contractile activity 2 h and 24 h after ET-1 addition. Surface area of cells/collagen lattices treated with DMSO and ET-1 was set to 1.0. Data are shown as mean ± SEM (*n* = 12); * statistically significant from DMSO/ET-1-treated cells (*p* < 0.01). (**B**) Immunofluorescence staining of murine HSCs using an antibody directed against the ET_A_R (in green) and Alexa-543 coupled wheat germ agglutinin (WGA, shown in red) to stain the cell surface. HSCs were incubated with either DMSO or a TGR5 agonist (10 μM) for 24 h prior to fixation and immunofluorescence staining. Colocalization of ET_A_R and WGA was determined using the coloc2 plugin for ImageJ. Colocalized pixels are shown in white in the merge images. (**C**) Colocalization was quantified using Pearson’s correlation coefficient for colocalization, as determined by the coloc2 plugin for ImageJ for each selected region of interest (ROI). Data are shown as mean ± SEM; * statistically significant from DMSO-treated cells (*n* = 8–9; 2–3 different ROIs per condition of three different cell isolations) (*p* < 0.05).

Activated HSCs are able to contract, leading to an increase in portal pressure due to their perivascular location in the space of Disse [40]. ET-1 has been identified as one of the key mediators regulating HSC contractility [24,41]. Since we observed an increase in portal pressure solely in TGR5 KO mice after LCA feeding, we hypothesized that activation and contraction of HSCs may contribute to this phenotype. To determine whether ET-1-dependent HSC contraction is modulated by TGR5, HSC contraction in response to ET-1 was analyzed. Rat HSCs were cultivated on collagen lattices for 7 days [40]. After this interval, DMSO, a TGR5 agonist (10 µM), or forskolin (10 µM) were added to the culture medium 30 min prior to stimulation with ET-1 (1 nM). Measurement of the collagen lattice surface area 2 h and 24 h after addition of ET-1 revealed a significantly smaller reduction in relative surface area in TGR5 agonist- or forskolin-treated cells, indicating an attenuation of HSC contractility (Figure 6A); 2 h: DMSO/ET-1 vs. TGR5 Ago/ET-1 1.0 vs. 1.64 ± 0.11 pixels^2^; DMSO/ET-1 vs. forskolin/ET-1 1.0 vs. 2.85 ± 0.36 pixels^2^, *n* = 12, *p* < 0.01; 24 h: DMSO/ET-1 vs. TGR5 Ago/ET-1 1.0 vs. 1.89 ± 0.27 pixels^2^; DMSO/ET-1 vs. forskolin/ET-1 1.0 vs. 3.96 ± 0.51, *n* = 12, *p* < 0.01. It was previously shown that cAMP may desensitize the ET-1 receptor-A (ET_A_R) towards its ligand on activated HSCs through internalization. Thus, the ET_A_R’s responsiveness is being shifted from picomolar to nanomolar concentrations [42]. Since TGR5 is coupled to a stimulatory G protein [6,8], we hypothesized that ligand binding to TGR5 could result in increased intracellular cAMP levels and internalization of the ET_A_R. Immunofluorescence staining localized the ET_A_R within the plasma membrane of HSCs, as demonstrated by colocalization with the plasma membrane marker wheat germ agglutinin (WGA) (Figure 6B). Treatment with the TGR5 agonist reduced the amount of ET_A_R within the plasma membrane, indicating internalization of the receptor (Figure 6C) (DMSO vs. TGR5 Ago (*n* = 8–9) 0.37 ± 0.06 Pearson’s correlation coefficient vs. 0.11 ± 0.04, 3.4-fold decrease, *p* < 0.01).

### 3.6. Activation of TGR5 Inhibits the ET-1-Mediated Increase in Portal Pressure

**Figure 7:**
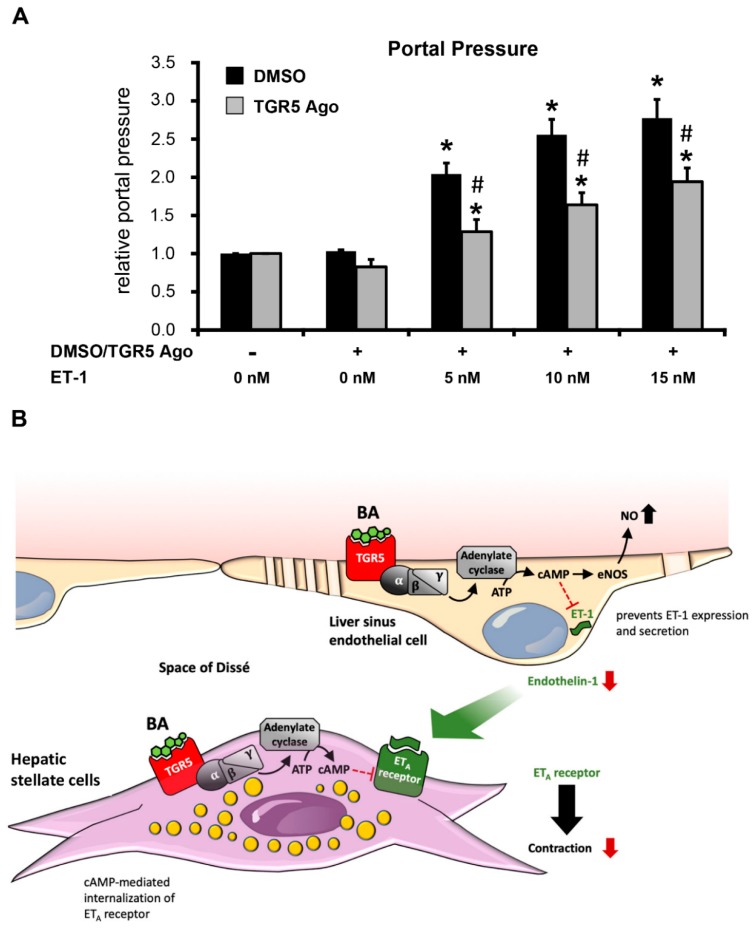
TGR5-dependent regulation of portal perfusion pressure. (**A**) Portal perfusion pressure of TGR5 transgenic mice (TGR5tg) was continuously measured in the presence of DMSO or a TGR5 agonist and increasing concentrations of ET-1. Perfusion was performed with either DMSO or a TGR5 agonist (10 μM) and increasing concentrations of endothelin-1 (ET-1, 5–15nM). Data are shown relative to controls, where the perfusion pressure in the presence of DMSO-containing perfusion buffer following an equilibrium phase of 15 min at the starting point was set to 1.0. All data are shown as mean ± SEM; * statistically significant from DMSO-treated cells (*p* < 0.05) (*n* = 6–9). (**B**) Cartoon depicting the mechanisms of TGR5-dependent inhibition of ET-1 signaling within the sinusoid. Activation of TGR5 through cAMP inhibits transcription and subsequent secretion of ET-1 from LSECs. ET-1, produced by LSECs, is known to induce contraction of activated HSCs cells by binding to the ET_A_R, leading to an increase of intrahepatic vascular resistance and, hence, the development of portal hypertension. Activation of TGR5 in HSCs promotes internalization of the ET_A_R, reducing ligand binding at the cell surface and thus contractile activity. In conclusion, ligand binding to TGR5 activates two mechanisms that act synergistically to reduce intrahepatic vascular resistance and, thus, portal hypertension.

Perfusion of mouse livers with buffer containing only DMSO as vehicle did not cause any significant changes in portal perfusion pressure. A challenge of these mice with increasing concentrations of ET-1 led to a dose-dependent increase in portal perfusion pressure. Addition of a TGR5 agonist to the ET-1-enriched perfusion buffer significantly reduced the ET-1-mediated rise in portal pressure as compared to vehicle containing perfusion buffer, indicating that TGR5 activation rapidly reduces ET-1-mediated portal hypertension (DMSO vs. TGR5 Ago/+1 nM ET-1 2.41 ± 0.26 cmH_2_0 vs. 1.42 ± 0.18 cmH_2_0 (1.7-fold decrease); DMSO vs. TGR5 Ago/+5 nM ET-1 2.9 ± 0.18 cmH_2_0 vs. 1.89 ± 0.28 cmH_2_0 (1.5-fold decrease), DMSO vs. TGR5 Ago/+15 nM ET-1 3.14 ± 0.15 cmH_2_0 vs. 2.20 ± 0.3 cmH_2_0 (1.4-fold decrease), (*n* = 5–6), *p* < 0.05) (Figure 7A).

## 4. Discussion

Liver injury of various etiologies triggers morphological and functional changes within hepatic sinusoids, resulting in development of fibrosis and portal hypertension [24,43,44]. Disturbances of the intercellular crosstalk within the sinusoids, especially between LSECs and HSCs, contribute to fibrosis development and microvascular dysfunction [24,43,44]. Multiple paracrine signals, such as NO, ET-1, TGF-β, PDGF, and VEGF, as well as various cytokines and chemokines that are being secreted not only from LSECs, hepatocytes, cholangiocytes, immune cells, but also from platelets, modulate LSEC and HSC phenotype under physiological and pathophysiological conditions [44,45]. Gene expression analysis of liver tissue following LCA feeding for 84 h revealed an enrichment in signaling pathways related to inflammation, proliferation, and matrix remodeling, which was in line with histological analysis of the livers. Interestingly, mice deficient for the G protein-coupled BA receptor TGR5 were more susceptible towards LCA-induced liver injury, resulting in elevated AST serum levels, more pronounced bile infarcts, and an elevated portal perfusion pressure. TGR5 is highly expressed in cholangiocytes, where activation of the receptor triggers formation of the bicarbonate umbrella, promotes tight junction integrity, and inhibits apoptosis, thus protecting cholangiocytes from bile acid toxicity [9,11,46,47]. Lack of TGR5 therefore renders mice more susceptible towards bile acid-induced biliary injury, as observed previously, in response to common bile duct ligation (CBDL) [19] and now in the LCA model. Furthermore, TGR5 exerts anti-inflammatory functions in monocytes and macrophages [4,6,13,14,18]. Absence of TGR5 resulted in elevated expression and secretion of chemokines and cytokines in response to lipopolysaccharide injection or common bile duct ligation [18,19]. Following LCA feeding, we observed a significant induction of hepatic mRNA expression of various cytokines, chemokines, and pro-fibrogenic chemokine receptors such as CCR1 and CCR5 [39]. Interestingly, expression of the chemokine receptor CCR5 was already induced 3-fold in TGR5 KO mice under chow-fed conditions, which has been previously reported for white adipose tissue in TGR5-deficient animals [13]. CXCL10 and its receptor CXCR3 have been implicated in fibrosis development in models of congenital hepatic fibrosis and carbon tetrachloride (CCl_4_)-mediated fibrosis [48,49]. While upregulation in comparison to chow-fed WT animals of CXCL10 was 9-fold in WT and 5-fold in TGR5 KO, respectively, CXCR3 mRNA expression was induced 3-fold in WT and 5-fold in TGR5 KO following LCA feeding. Interestingly, in patients with chronic liver disease, serum CXCL10 levels, but not hepatic CXCL10 mRNA levels, were positively correlated with portal hypertension, fibrosis stage, and disease progression [50,51]. To which extent the CXCL10–CXCR3 signaling pathway contributes to the phenotypes of our LCA-fed mice is unclear. CXCL1, which is secreted from LSECs following mechanical stretching, triggers formation of sinusoidal microthrombi, thereby increasing portal pressure independent of cirrhosis [52]. Hepatic CXCL1 mRNA expression was significantly induced in LCA-fed mice. In comparison to chow-fed WT mice, CXCL1 levels were 14.5- and 9.4-fold higher in LCA-fed WT and TGR5 KO mice, respectively. However, in TGR5 KO mice, CXCL1 levels were low under chow-fed conditions and increased 27-fold in response to LCA. Thus, microthrombosis may also contribute to portal hypertension in LCA-fed TGR5 KO animals.

Moreover, expression of PDGFRα/β, TGF-β1 and ET-1, which are related to HSC activation, development of fibrosis, and portal hypertension, were upregulated in both genotypes in response to LCA. However, mRNA levels were significantly higher in livers of TGR5 KO mice as compared to liver tissue from WT littermates. While periportal fibrosis, as measured by Sirius red staining and hydroxyproline content, was not significantly increased in either genotype as early as 3.5 days after starting the LCA feeding, expression of collagen was significantly upregulated in both genotypes, suggesting initiation of fibrogenesis in line with previous data [21]. Since TGR5 is expressed both in LSECs and activated HSCs, we further explored TGR5-mediated effects in these cell types. Stimulation of TGR5 on LSECs reduced ET-1 expression, as well as its secretion, and reduced the responsiveness of activated HSCs towards ET-1 through internalization of the ET_A_R. These two mechanisms may act synergistically to reduce ET-1 signaling within the sinusoid (Figure 7B). In rats, treatment with a TGR5 agonist (BAR501) for 6 days prior to cannulation of the portal vein lowered the rise in portal perfusion pressure in response to norepinephrine [53]. Furthermore, administration of the TGR5 agonist inhibited portal hypertension in mice treated for 9 weeks with CCl_4_, while it did not affect fibrosis development [53], suggesting an effect on the hepatic microvasculature independent of extracellular matrix deposition. As underlying molecular mechanisms, the authors demonstrated that TGR5 activation induces expression and also non-genomically promotes activation of cystathione- γ-lyase (CSE), resulting in increased production of hydrogen sulfide (H_2_S), a potent vasodilator [53,54]. Amongst others, we have previously shown that TGR5 may also trigger serine phosphorylation of eNOS, thereby promoting NO generation, which again leads to vasodilation of hepatic sinusoids [1,53]. Taken together, TGR5 agonists promote generation and secretion of vasodilatory agents (H_2_S and NO) and inhibit expression and secretion of the potent vasoconstrictor ET-1 from LSECs [1,7,46,53,54]. While the function of TGR5 in LSECs has been previously studied, the role of TGR5 for HSC activation and function remained elusive. Interestingly, the rise in hepatic PDGFRα/β expression was more pronounced in TGR5 KO mice as compared to WT littermates after LCA-feeding. PDGF-β, which may be derived from either LSECs or platelets, can signal to activated HSCs through its receptor PDGFRβ and promote cell proliferation, migration, and development of a pro-angiogenic HSC phenotype [55,56]. Furthermore, it was recently demonstrated that PDGF-β plays a role in the trans-differentiation processes of HSCs into an activated phenotype, and that administration of an anti-PDGF-β antibody (MPR8457) inhibits development and progression of biliary fibrosis in Abcb4 KO mice, which serve as a chronic model for sclerosing cholangitis [56,57]. Moreover, cAMP may desensitize activated HSCs towards ET-1 through internalization of the ET_A_R [42]. Using a non-BA TGR5 ligand, we could demonstrate that activation of the receptor promotes retrieval of ET_A_R from the plasma membrane, explaining the reduced ET-1-dependent contractility of activated HSCs in the presence of a TGR5 agonist in vitro. In summary, TGR5 activation reduces hepatic vascular resistance through several mechanisms, both in LSECs and in HSCs, that act synergistically. Through modulation of PDGFRβ expression, it may also contribute to the response of HSCs to microvascular thrombosis and platelet-derived signals. A recent study explored the effect of the non-bile acid TGR5 ligand oleanolic acid in a rat model of liver fibrosis development [58]. Treatment with oleanolic acid significantly reduced fibrogenesis in vivo; however, a direct effect on HSCs in vitro was not observed, since the cell lines used did not express TGR5 [58]. It was previously reported that TGR5 expression, which is very low in quiescent HSCs, is upregulated during the activation of HSCs into a myofibroblast-like phenotype [4,26]. Thus, stimulation of TGR5 on activated HSCs may contribute to the beneficial effects of oleanolic acid on fibrosis development in the above model [58]. Furthermore, mice deficient in both TGR5 and the nuclear bile acid receptor FXR were generated and showed an enrichment in gene expression pathways associated with liver fibrosis and inflammation in line with our study [59]. Since FXR agonists convey beneficial effects in preclinical models of liver fibrosis and portal hypertension [60], it is highly anticipated that TGR5/FXR double-KO mice will also develop LSEC and HSC dysfunction and portal hypertension. Thus, targeting of BA signaling becomes an interesting strategy to reverse morphological changes and dysfunction of cell types residing in the hepatic sinusoids, thereby attenuating portal hypertension and fibrosis development.

## Figures and Tables

**Table 1:** Selection of genes significantly regulated in livers of TGR5 wildtype (WT) and TGR5 knockout (KO) after LCA feeding for 84 h as compared to chow-fed animals of the same genotypes. Data were generated by real-time PCR, as described in Section 2—Materials and Methods. Gene expression is presented in relation to the house-keeping gene SDHA. Values for WT mice on chow diet were set to 1.0. * Significantly different as compared to chow-fed animals of the same genotype (*p* < 0.05); ** significantly different as compared to chow-fed animals of the same genotype (*p* < 0.01); # significantly different as compared to WT animals of the same treatment group (*p* < 0.05). All data are expressed as mean ± SEM. Green and red boxes indicate a statistically significant downregulation or upregulation, respectively, of a gene in TGR5 KO as compared to WT littermates receiving the same treatment.

	WT Chow		WT LCA		WT LCA		KO LCA	
	Mean	SEM	MW	SEM	MW	SEM	MW	SEM
Col1α1	1.00	0.57	1.71	0.81	65.77 **	18.57	68.82 **	13.78
Col1α2	1.00	0.35	1.69	0.39	25.59 **	6.53	21.67 **	3.39
PAI-1	1.00	0.27	0.92	0.31	120.21 **	24.53	160.23 **	29.05
TIMP-1	1.00	0.44	4.03	2.74	492.23 *	159.09	456.28 **	93.63
VEGF-C	1.00	0.21	2.00	0.50	5.26 *	1.69	7.23 *	1.64
eNOS	1.00	0.26	1.43	0.46	10.07 **	1.39	10.19 **	1.46
VAP-1	1.00	0.70	0.40	0.12	5.78 *	1.98	4.92 **	1.03
CD163	1.00	0.23	1.50	0.13	16.23 **	4.64	16.53 **	3.12
CCR1	1.00	0.55	1.89	0.50	134.81 **	32.27	111.67 **	21.33
CCR5	1.00	0.37	3.16 #	0.26	35.52 **	8.26	17.20 **#	3.18
CXCR3	1.00	0.20	1.57	0.42	3.11 **	0.67	5.35 **#	0.67
CXCR4	1.00	0.37	0.83	0.22	14.53 **	3.53	13.41 **	2.68
CXCR7	1.00	0.45	1.32	0.38	28.23 **	7.24	26.56 **	5.45
CCL2	1.00	0.50	1.40	0.80	293.80 **	30.39	384.99 **	71.68
CCL3	1.00	0.50	3.46	1.58	98.31 **	18.18	95.45 **	15.64
CCL4	1.00	0.76	1.26	0.37	55.92 **	12.79	38.48 **	6.40
CCL25	1.00	0.28	2.14	0.20	3.16 **	0.34	3.13 **	0.34
CXCL1	1.00	0.76	0.35	0.07	14.48 **	1.64	9.38 **#	1.00
CXCL10	1.00	0.17	1.22	0.42	9.69 **	1.95	5.03 **#	0.68
IL1β	1.00	0.20	1.91	0.44	16.34 **	2.91	17.07 **	2.41
IL10	1.00	0.08	1.24	0.32	26.43 **	4.13	48.14 **	12.76
TNFα	1.00	0.59	2.19	0.94	19.78 **	3.49	21.39 **	3.35
TGF-β2	1.00	0.50	1.00	0.32	16.55 **	2.86	16.95 **	2.71
TGFβR1	1.00	0.06	1.66	0.50	7.99 **	0.94	8.84 **	0.92
CyclinD1	1.00	0.31	1.36	0.35	17.57 **	2.81	8.95 **#	0.98
SOX9	1.00	0.17	1.24	0.06	16.03 **	2.89	16.02 **	1.00
Caspase3	1.00	0.20	1.65	0.20	10.92 **	1.98	10.46 **	1.38
RIPK1	1.00	0.14	1.28	0.12	4.00 **	0.49	4.32 **	0.50
RIPK3	1.00	0.27	1.89	0.45	21.74 **	2.72	28.57 **	3.27
MT-1	1.00	0.48	1.15	0.36	140.44 **	40.83	104.00 **	28.48
MT-2	1.00	0.38	1.21	0.59	293.15 *	97.95	186.38 **	46.39
CYP7A1	1.00	0.22	2.23	0.57	0.01 **	0.00	0.01 **	0.00
CYP8B1	1.00	0.28	1.20	0.26	0.01 *	0.00	0.01 *	0.00

## Supplementary Materials

The following are available online at https://www.mdpi.com/2073-4409/8/11/1467/s1, Figure S1: Expression of TGR5 in rodent HSCs Figure S2: HSC contraction assay Table S1: Differentially upregulated genes LCA vs. chow; Table S2: Differentially downregulated genes LCA vs. chow; Table S3: Enriched GO pathways LCA vs. chow; Table S4: Downregulated GO pathways LCA vs. chow.

## References

[1] Keitel V., Reinehr R., Gatsios P., Rupprecht C., Görg B., Selbach O., Häussinger D., Kubitz R. (2007). The G-protein coupled bile salt receptor TGR5 is expressed in liver sinusoidal endothelial cells. Hepatology.

[2] Pols T.W., Nomura M., Harach T., Lo Sasso G., Oosterveer M.H., Thomas C., Rizzo G., Gioiello A., Adorini L., Pellicciari R. (2011). TGR5 activation inhibits atherosclerosis by reducing macrophage inflammation and lipid loading. Cell Metab..

[3] Deutschmann K., Reich M., Klindt C., Droge C., Spomer L., Häussinger D., Keitel V. (2018). Bile acid receptors in the biliary tree: TGR5 in physiology and disease. Biochim. Biophys. Acta Mol. Basis Dis..

[4] Keitel V., Donner M., Winandy S., Kubitz R., Häussinger D. (2008). Expression and function of the bile acid receptor TGR5 in Kupffer cells. Biochem. Biophys. Res. Commun..

[5] Kordes C., Sawitza I., Götze S., Häussinger D. (2015). Bile acids and stellate cells. Dig. Dis..

[6] Kawamata Y., Fujii R., Hosoya M., Harada M., Yoshida H., Miwa M., Fukusumi S., Habata Y., Itoh T., Shintani Y. (2003). AG protein-coupled receptor responsive to bile acids. J. Biol. Chem..

[7] Keitel V., Stindt J., Häussinger D. (2019). Bile Acid-Activated Receptors: GPBAR1 (TGR5) and Other G Protein-Coupled Receptors. Handb. Exp. Pharmacol..

[8] Maruyama T., Miyamoto Y., Nakamura T., Tamai Y., Okada H., Sugiyama E., Nakamura T., Itadani H., Tanaka K. (2002). Identification of membrane-type receptor for bile acids (M-BAR). Biochem. Biophys. Res. Commun..

[9] Keitel V., Cupisti K., Ullmer C., Knoefel W.T., Kubitz R., Häussinger D. (2009). The membrane-bound bile acid receptor TGR5 is localized in the epithelium of human gallbladders. Hepatology.

[10] Keitel V., Reich M., Häussinger D. (2015). TGR5: Pathogenetic role and/or therapeutic target in fibrosing cholangitis?. Clin. Rev. Allergy Immunol..

[11] Merlen G., Kahale N., Ursic-Bedoya J., Bidault-Jourdainne V., Simerabet H., Doignon I., Tanfin Z., Garcin I., Péan N., Gautherot J. (2019). TGR5-dependent hepatoprotection through the regulation of biliary epithelium barrier function. Gut.

[12] Perino A., Schoonjans K. (2015). TGR5 and Immunometabolism: Insights from Physiology and Pharmacology. Trends Pharmacol. Sci..

[13] Perino A., Pols T.W., Nomura M., Stein S., Pellicciari R., Schoonjans K. (2014). TGR5 reduces macrophage migration through mTOR-induced C/EBPbeta differential translation. J. Clin. Investig..

[14] Guo C., Xie S., Chi Z., Zhang J., Liu Y., Zhang L., Zheng M., Zhang X., Xia D., Ke Y. (2016). Bile Acids Control Inflammation and Metabolic Disorder through Inhibition of NLRP3 Inflammasome. Immunity.

[15] Vassileva G., Golovko A., Markowitz L., Abbondanzo S.J., Zeng M., Yang S., Hoos L., Tetzloff G., Levitan D., Murgolo N.J. (2006). Targeted deletion of Gpbar1 protects mice from cholesterol gallstone formation. Biochem. J..

[16] Maruyama T., Tanaka K., Suzuki J., Miyoshi H., Harada N., Nakamura T., Miyamoto Y., Kanatani A., Tamai Y. (2006). Targeted disruption of G protein-coupled bile acid receptor 1 (Gpbar1/M-Bar) in mice. J. Endocrinol..

[17] Pean N., Doignon I., Garcin I., Besnard A., Julien B., Liu B., Branchereau S., Spraul A., Guettier C., Humbert L. (2013). The receptor TGR5 protects the liver from bile acid overload during liver regeneration in mice. Hepatology.

[18] Wang Y.D., Chen W.D., Yu D., Forman B.M., Huang W. (2011). The G-protein-coupled bile acid receptor, Gpbar1 (TGR5), negatively regulates hepatic inflammatory response through antagonizing nuclear factor kappa light-chain enhancer of activated B cells (NF-kappaB) in mice. Hepatology.

[19] Reich M., Deutschmann K., Sommerfeld A., Klindt C., Kluge S., Kubitz R., Ullmer C., Knoefel W.T., Herebian D., Mayatepek E. (2016). TGR5 is essential for bile acid-dependent cholangiocyte proliferation in vivo and in vitro. Gut.

[20] Sato M., Sato K., Furuse M. (2008). Change in hepatic and plasma bile acid contents and its regulatory gene expression in the chicken embryo. Comp. Biochem. Physiol. B Biochem. Mol. Biol..

[21] Fickert P., Fuchsbichler A., Marschall H.U., Wagner M., Zollner G., Krause R., Zatloukal K., Jaeschke H., Denk H., Trauner M. (2006). Lithocholic acid feeding induces segmental bile duct obstruction and destructive cholangitis in mice. Am. J. Pathol..

[22] Woolbright B.L., Li F., Xie Y., Farhood A., Fickert P., Trauner M., Jaeschke H. (2014). Lithocholic acid feeding results in direct hepato-toxicity independent of neutrophil function in mice. Toxicol. Lett..

[23] Pimpin L., Cortez-Pinto H., Negro F., Corbould E., Lazarus J.V., Webber L., Sheron N., EASL HEPAHEALTH Steering Committee (2018). Burden of liver disease in Europe: Epidemiology and analysis of risk factors to identify prevention policies. J. Hepatol..

[24] McConnell M., Iwakiri Y. (2018). Biology of portal hypertension. Hepatol. Int..

[25] Iwakiri Y., Shah V., Rockey D.C. (2014). Vascular pathobiology in chronic liver disease and cirrhosis—Current status and future directions. J. Hepatol..

[26] Sawitza I., Kordes C., Götze S., Herebian D., Häussinger D. (2015). Bile acids induce hepatic differentiation of mesenchymal stem cells. Sci. Rep..

[27] Ullmer C., Alvarez Sanchez R., Sprecher U., Raab S., Mattei P., Dehmlow H., Sewing S., Iglesias A., Beauchamp J., Conde-Knape K. (2013). Systemic bile acid sensing by G protein-coupled bile acid receptor 1 (GPBAR1) promotes PYY and GLP-1 release. Br. J. Pharmacol..

[28] Thomas C., Gioiello A., Noriega L., Strehle A., Oury J., Rizzo G., Macchiarulo A., Yamamoto H., Mataki C., Pruzanski M. (2009). TGR5-mediated bile acid sensing controls glucose homeostasis. Cell Metab..

[29] Vassileva G., Hu W., Hoos L., Tetzloff G., Yang S., Liu L., Kang L., Davis H.R., Hedrick J.A., Lan H. (2010). Gender-dependent effect of Gpbar1 genetic deletion on the metabolic profiles of diet-induced obese mice. J. Endocrinol..

[30] Jamall I.S., Finelli V.N., Que Hee S.S. (1981). A simple method to determine nanogram levels of 4-hydroxyproline in biological tissues. Anal. Biochem..

[31] Bartneck M., Topuz F., Tag C.G., Sauer-Lehnen S., Warzecha K.T., Trautwein C., Weiskirchen R., Tacke F. (2015). Molecular response of liver sinusoidal endothelial cells on hydrogels. Mater. Sci. Eng. C Mater. Biol. Appl..

[32] Kordes C., Sawitza I., Müller-Marbach A., Ale-Agha N., Keitel V., Klonowski-Stumpe H., Häussinger D. (2007). CD133+ hepatic stellate cells are progenitor cells. Biochem. Biophys. Res. Commun..

[33] Schneider C.A., Rasband W.S., Eliceiri K.W. (2012). NIH Image to ImageJ: 25 years of image analysis. Nat. Methods.

[34] Schindelin J., Arganda-Carreras I., Frise E., Kaynig V., Longair M., Pietzsch T., Preibisch S., Rueden C., Saalfeld S., Schmid B. (2012). Fiji: An open-source platform for biological-image analysis. Nat. Methods.

[35] Chazotte B. (2011). Labeling membrane glycoproteins or glycolipids with fluorescent wheat germ agglutinin. Cold Spring Harb. Protoc..

[36] Smyth G.K. (2005). Limma: Linear Models for Microarray Data. Bioinformatics and Computational Biology Solutions Using R and Bioconductor.

[37] Benjamini Y., Hochberg Y. (1995). Controlling the False Discovery Rate: A Practical and Powerful Approach to Multiple Testing. J. R. Stat. Soc. Ser. B Methodol..

[38] Alexa A., Rahnenführer J., Lengauer T. (2006). Improved scoring of functional groups from gene expression data by decorrelating GO graph structure. Bioinformatics.

[39] Seki E., De Minicis S., Gwak G.Y., Kluwe J., Inokuchi S., Bursill C.A., Llovet J.M., Brenner D.A., Schwabe R.F. (2009). CCR1 and CCR5 promote hepatic fibrosis in mice. J. Clin. Investig..

[40] Rockey D.C., Housset C.N., Friedman S.L. (1993). Activation-dependent contractility of rat hepatic lipocytes in culture and in vivo. J. Clin. Investig..

[41] Iwakiri Y. (2012). Endothelial dysfunction in the regulation of cirrhosis and portal hypertension. Liver Int..

[42] Reinehr R., Fischer R., Häussinger D. (2002). Regulation of endothelin-A receptor sensitivity by cyclic adenosine monophosphate in rat hepatic stellate cells. Hepatology.

[43] Greuter T., Shah V.H. (2016). Hepatic sinusoids in liver injury, inflammation, and fibrosis: New pathophysiological insights. J. Gastroenterol..

[44] Marrone G., Shah V.H., Gracia-Sancho J. (2016). Sinusoidal communication in liver fibrosis and regeneration. J. Hepatol..

[45] Lee Y.A., Wallace M.C., Friedman S.L. (2015). Pathobiology of liver fibrosis: A translational success story. Gut.

[46] Keitel V., Häussinger D. (2018). Role of TGR5 (GPBAR1) in Liver Disease. Semin. Liver Dis..

[47] Beuers U., Hohenester S., de Buy Wenniger L.J., Kremer A.E., Jansen P.L., Elferink R.P. (2010). The biliary HCO(3)(-) umbrella: A unifying hypothesis on pathogenetic and therapeutic aspects of fibrosing cholangiopathies. Hepatology.

[48] Hintermann E., Bayer M., Pfeilschifter J.M., Luster A.D., Christen U. (2010). CXCL10 promotes liver fibrosis by prevention of NK cell mediated hepatic stellate cell inactivation. J. Autoimmun..

[49] Kaffe E., Fiorotto R., Pellegrino F., Mariotti V., Amenduni M., Cadamuro M., Fabris L., Strazzabosco M., Spirli C. (2018). beta-Catenin and interleukin-1beta-dependent chemokine (C-X-C motif) ligand 10 production drives progression of disease in a mouse model of congenital hepatic fibrosis. Hepatology.

[50] Lehmann J.M., Claus K., Jansen C., Pohlmann A., Schierwagen R., Meyer C., Thomas D., Manekeller S., Claria J., Strassburg C.P. (2018). Circulating CXCL10 in cirrhotic portal hypertension might reflect systemic inflammation and predict ACLF and mortality. Liver Int..

[51] Tacke F., Zimmermann H.W., Berres M.L., Trautwein C., Wasmuth H.E. (2011). Serum chemokine receptor CXCR3 ligands are associated with progression, organ dysfunction and complications of chronic liver diseases. Liver Int..

[52] Hilscher M.B., Sehrawat T., Arab J.P., Zeng Z., Gao J., Liu M., Kostallari E., Gao Y., Simonetto D.A., Yaqoob U. (2019). Mechanical Stretch Increases Expression of CXCL1 in Liver Sinusoidal Endothelial Cells to Recruit Neutrophils, Generate Sinusoidal Microthombi, and Promote Portal Hypertension. Gastroenterology.

[53] Renga B., Cipriani S., Carino A., Simonetti M., Zampella A., Fiorucci S. (2015). Reversal of Endothelial Dysfunction by GPBAR1 Agonism in Portal Hypertension Involves a AKT/FOXOA1 Dependent Regulation of H2S Generation and Endothelin-1. PLoS ONE.

[54] Renga B., Bucci M., Cipriani S., Carino A., Monti M.C., Zampella A., Gargiulo A., d‘Emmanuele di Villa Bianca R., Distrutti E., Fiorucci S. (2015). Cystathionine gamma-lyase, a H2S-generating enzyme, is a GPBAR1-regulated gene and contributes to vasodilation caused by secondary bile acids. Am. J. Physiol. Heart Circ. Physiol..

[55] Semela D., Das A., Langer D., Kang N., Leof E., Shah V. (2008). Platelet-derived growth factor signaling through ephrin-b2 regulates hepatic vascular structure and function. Gastroenterology.

[56] Yoshida S., Ikenaga N., Liu S.B., Peng Z.W., Chung J., Sverdlov D.Y., Miyamoto M., Kim Y.O., Ogawa S., Arch R.H. (2014). Extrahepatic platelet-derived growth factor-beta, delivered by platelets, promotes activation of hepatic stellate cells and biliary fibrosis in mice. Gastroenterology.

[57] Fickert P., Pollheimer M.J., Beuers U., Lackner C., Hirschfield G., Housset C., Keitel V., Schramm C., Marschall H.U., Karlsen T.H. (2014). Characterization of animal models for primary sclerosing cholangitis (PSC). J. Hepatol..

[58] Kaya D., Kaji K., Tsuji Y., Yamashita S., Kitagawa K., Ozutsumi T., Fujinaga Y., Takaya H., Kawaratani H., Moriya K. (2019). TGR5 Activation Modulates an Inhibitory Effect on Liver Fibrosis Development Mediated by Anagliptin in Diabetic Rats. Cells.

[59] Ferrell J.M., Pathak P., Boehme S., Gilliland T., Chiang J.Y.L. (2019). Deficiency of Both Farnesoid X Receptor and Takeda G Protein-Coupled Receptor 5 Exacerbated Liver Fibrosis in Mice. Hepatology.

[60] Schwabl P., Hambruch E., Seeland B.A., Hayden H., Wagner M., Garnys L., Strobel B., Schubert T.L., Riedl F., Mitteregger D. (2017). The FXR agonist PX20606 ameliorates portal hypertension by targeting vascular remodelling and sinusoidal dysfunction. J. Hepatol..

