# Supplementary material for: The G Protein-Coupled Bile Acid Receptor TGR5 (Gpbar1) Modulates Endothelin-1 Signaling in Liver
Source: Cells. 2019 Nov 19;8(11):1467. doi: 10.3390/cells8111467 (PMC6912679; doi:10.3390/cells8111467)
Supplement: Supplementary file 1 [file cells-08-01467-s001.zip › Supplemental Figures.pdf]

A

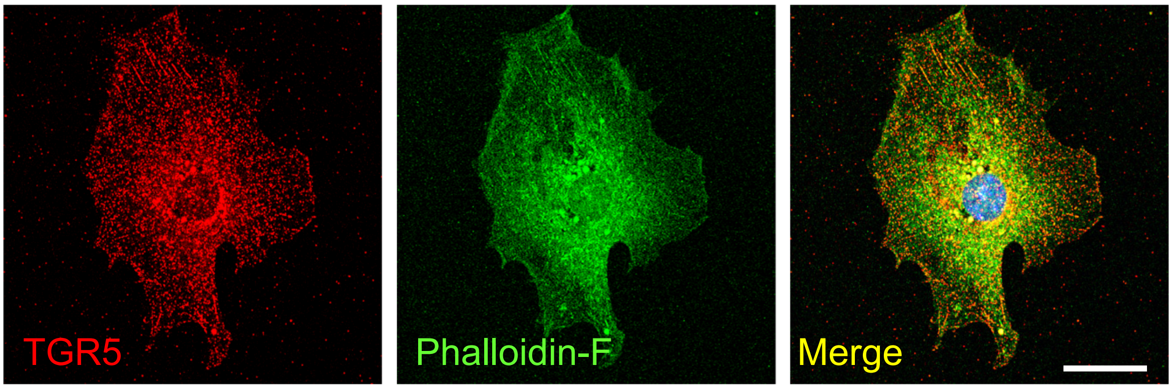

B

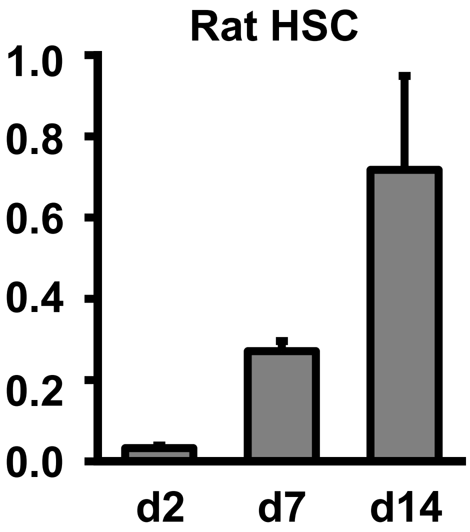

Supplemental Figure 1: Activated rodent HSCs express TGR5. A: Representative immunofluorescence staining of actived murine HSCs for TGR5 (Cy3, red) and Phalloidin-Fitc (Phalloidin-F, shown in green). Cells were cultured for 7 days on plastic dishes, which leads to differentiation into a myofibroblast-like phenotype. Images demonstrate localization of the receptor within the plasma membrane as well as in intracellular vesicular structures. Bar=10µm. B: Semiquantitative realtime PCR analysis of rat HSCs at day 2 (d2), day 7 (d7) and day 14 (d14) after isolation and cultivation on plastic dishes demonstrates an upregulation of TGR5 expression in activated HSCs. Data are presented as mean±SEM (n=4-5).

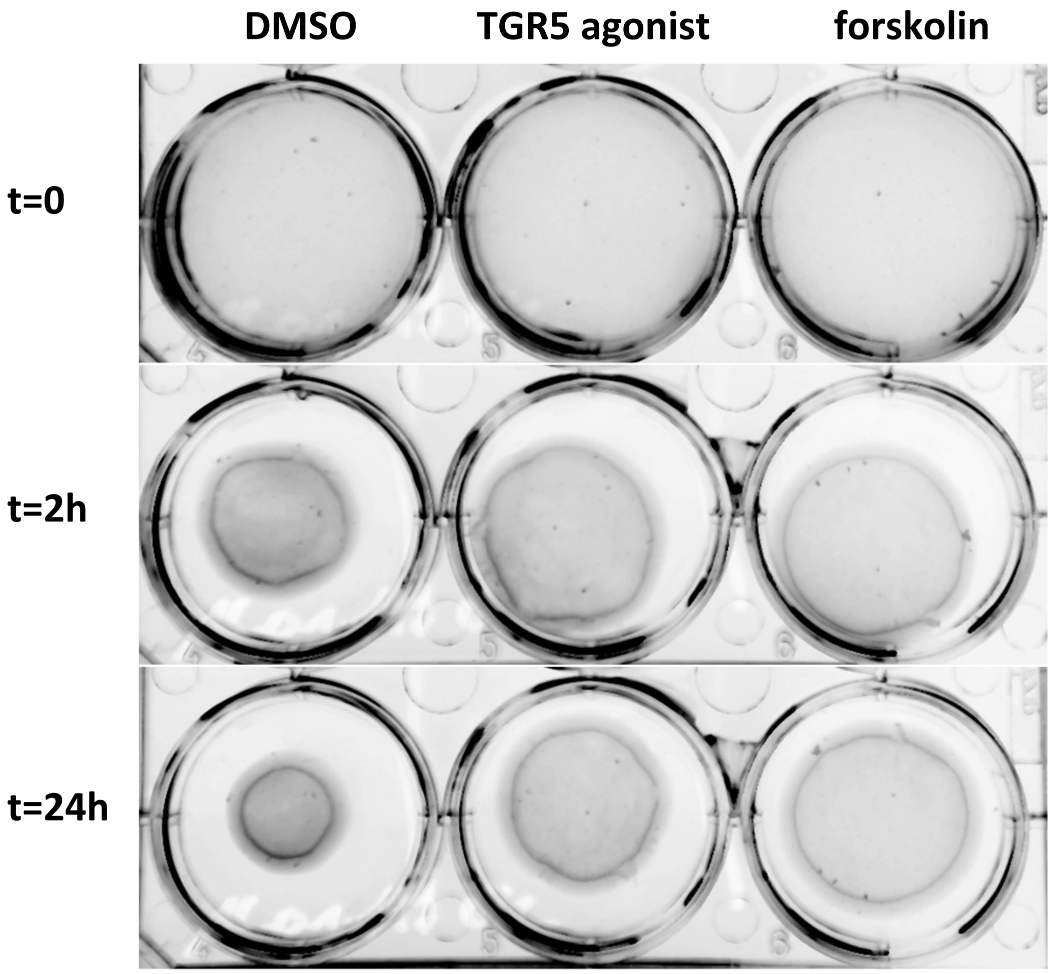

Supplemental Figure 2: Representative image of the HSC contraction assay. HSCs were cultured for 7 days on thick collagen lattices. After 7 days cells were treated with DMSO, a TGR5 agonist (10µM) or forskolin (10µM) for 30min. After this pre-incubation, cells were treated with ET-1 and lattices were detached from the cell culture dishes. Pictures were taken at time of ET-1 application as well as 2h and 24h after stimulation with ET-1. The size of the collagen lattices for each time point was determined using the ImageJ software as described in the materials and methods section.
